# Extracellular vesicles derived from different sources play various roles in diabetic retinopathy

**DOI:** 10.3389/fendo.2022.1064415

**Published:** 2023-01-04

**Authors:** Tingting Chen, Fang Wang, Jiayi Wei, Le Feng

**Affiliations:** Department of Ophthalmology, Shanghai Tenth People’s Hospital Affiliated to Tongji University School of Medicine, Shanghai, China

**Keywords:** diabetic retinopathy, extracellular vesicles (EVs), exosomes, biomarker, treatment

## Abstract

Extracellular vesicles (EVs) are present in almost all biological fluids and secreted by almost all cell types. A growing number of studies have revealed the potential roles of EVs in the diagnosis and treatment of the diabetic retinopathy (DR). Changes in the quantity and content of EVs may serve as biomarkers of cause or consequence of pathological status of DR, such as inflammation, neovascularization and epithelial-mesenchymal transition. In addition, as natural, safe and efficient drug carrier, EVs have been reported to play important roles in intercellular communication by acting for essential cell-specific information to target cells. In this review, we summarize the roles of EVs, secreted by various types of cells and participated in various biological processes, in the pathogenesis, diagnosis, and treatment of DR.

## 1 Introduction

Diabetic retinopathy (DR) is one of the most common and serious complications of diabetes, which remains the most prevalent cause of visual impairment in the working-age adults, eventually resulting in irreversible vision loss. DR can be divided into non-proliferative DR (NPDR) and proliferative DR (PDR) according to the absence or presence of retinal neovascularization. Severe visual impairment and blindness may due to the late pathological events of PDR like vitreous hemorrhage, traction retinal detachment and neovascular glaucoma (1). It is worth mentioning that diabetic macular edema (DME) can appear in any stage of DR and has surpassed PDR as the leading cause of visual impairment in patients (2). At present, the treatment modalities for DR include laser photocoagulation, intraocular injections of anti-VEGF or steroids and vitrectomy (3–5). However, these can not completely prevent progression of the disease and reverse visual impairment. In recent years, the prevalence of DR has been increasing, but the early diagnosis and treatment of DR are limited. When patients perceive changes in vision, the visual impairment is irreversible (6). Therefore, it is of great significance to improve the understanding of pathogenesis in DR, and looking for early diagnosis and treatment methods for preventing DR-related visual impairment.

Extracellular vesicles (EVs) which include ectosomes and exosomes, are membrane-derived vesicles that mediate intercellular communications (7). Ectosomes are formed by outward blebbing from the plasma membrane, which include microvesicles, microparticles, and large vesicles ranging from approximately 50 nm to 1 μm in diameter, while exosomes are endosomal origin ranging in size from 40 to 160 nm in diameter (8). EVs are extracellularly released from the majority of human cell types and have been isolated from most bodily fluids, such as vitreous fluid, blood, urine, saliva, breast milk, amniotic fluid, ascites, cerebrospinal fluid, bile and semen etc. (9, 10). The enrichment of tetraspanins such as CD6, CD81, CD9, CD82, CD53 and CD37 can be used as EVs markers (11, 12). EVs are known to contain proteins, lipids, and nucleic acids, which may change in different states of the extracellular environment or secreting cells. Of note, miRNA in nucleic acids are exported out of the cell and can influence gene expression in distant cells (9). Therefore structural and functional properties enable EVs with the characteristics of high specificity, high sensitivity and targeted transport, which are potentially valuable in diagnostic and therapeutic applications.

In recent years, many researches have shown that EVs also play important role in the diagnosis and treatment of DR. In this review, we have summarized EVs, from different sources like intraocular cell-derived EVs, mesenchymal stem cell (MSC)-derived EVs and blood-derived EVs, work in different pathways in DR, so that we can provide new ideas for the study of pathogenesis in DR, and look for early diagnosis and treatment.

## 2 EVs that play roles in the pathogenesis/diagnosis/prognosis of DR

### 2.1 Intraocular cell-derived EVs

Endothelial cells (ECs), pericytes, astrocytes in the retina (RAC) and retinal pigment epithelial (RPE) cells are important components of inner and outer blood-retinal barrier (BRBs), and play important roles in maintaining the homeostasis of the retinal microenvironment (13). RAC is strictly related to retinal blood vessels and the main producer of VEGF in both normal and pathological angiogenesis (14). Normal pericytes provide vessel stability and inhibit the proliferation and migration of vascular ECs. The loss of pericytes lead not only to the vasodynamic changes in the early stage of DR, but also to the neovascularization in PDR (15). As DR progresses, the tight junction complexes between RPE cells are disassemble and the pathological leakage occurs in paracellular space (13). When BRB is destroyed, retinal microglia and complement system can maintain retinal homeostasis by regulating the immune system (16). The proliferation and migration of RPE cells and the secretion of extracellular matrix molecules contribute to the formation of fibrotic membranes in the advanced stage of DR, PDR (17). The characteristics of EVs can generally reflect the phenotype and physiology of the parent cell. EVs of different retinal cells have different proteins or genetic information, which can change under pathophysiological conditions, and these minor changes can eventually lead to the retinopathy (18).

#### 2.1.1 Retinal pigment epithelial cell-derived EVs

RPE-derived EVs are involved in the regulation of oxidative stress, inflammation, apoptosis and neovascularization during DR progression. In addition, endothelial-to-mesenchymal transition (EndoMT) lead to pathological fibrosis in PDR, and RPE-derived EVs play potential roles to inhibit EndoMT

Atienzar-Aroca, S et al. (19) found that EVs derived from healthy RPE cell inhibit blood vessel formation under physiological conditions, while those released from stressed ARPE‐19 cells promote angiogenesis. This angiogenic effect might be because of the extra cargo of proteins and mRNA contained in the EVs and HUVEC cells influenced by them showed higher levels of VEGF receptors. The further research proposed that the abnormal vessel growth correlate with augmented VEGFR2‐expressing EVs, which released from stressed ARPE‐19 cells and directly associated with autophagy, causing ECs to migrate and angiogenesis (20). Maisto, R et al. (21) showed that VEGF-containing EVs, which released by ARPE-19 and promoted neovascularization in HUVECs, can be reversed by the melanocortin 5 receptor agonist (MCR5) activation. Ke, Y et al. (22) demonstrated that RPE-derived EVs under oxidative stress increased apoptosis, induced oxidative damage and inflammatory response through Apaf1/caspase-9 axis. Meanwhile, Gu, S et al. (23) proposed that EVs from ARPE-19 cells may transmit miR-202-5p through TGF/Smad pathway, which is an important mediator for intercellular crosstalk to suppress EndoMT and may be a potential treatment for PDR pathological fibrosis.

#### 2.1.2 Pericyte-derived EVs

Pericyte loss is considered a hallmark of early DR, and the changes in pericyte-derived EVs in diabetic conditions may also be of potential value as early biomarkers of DR. Moreover, pericyte-derived Vs still play roles in the crosstalk between pericytes and ECs, which is essential for vessel stabilization and remodeling.

Study by Mazzeo, A et al. (24) showed that miR-126 expression is down-regulated pericyte-derived EVs in diabetic-like conditions, which involved in vessel destabilization in DR. Unfortunately, they did not find PDGF and Ang-2 signalling pathways associated with this mechanism. In addition, Liu, C et al. (25) found that diabetes-related stress up-regulated the expression of mmu_circ_0000254 in pericytes and named it cPWWP2A. cPWWP2A directly regulates pericytes biology but indirectly regulates ECs biology *via* EVs carrying cPWWP2A, which inhibited miR-579 activity, increased expression of angiopoietin 1/occludin/SIRT1, and ultimately alleviates diabetes-induced retinal vascular dysfunction.

#### 2.1.3 Retinal astrocytes-derived EVs

RAC-derived EVs are involved in the regulation of endothelial function. Study by Hajrasouliha, A. R et al. (26) showed that RAC-derived EVs inhibited retinal vessel leakage and choroidal neovascularization by targetting both macrophages and ECs. They reduced the number of infiltrating macrophages, directly antagonized the inflammatory and angiogenic factors, and inhibited the migration and vascular tubule forming of ECs. Zhu, L et al. (27) found that RAC-derived EVs under normal and oxidative stress conditions had different effects on the endothelial function, which can be reversed by the exosome inhibitor GW4869 or the autophagy inhibitor 3-methyladenine. This study indicated that oxidative stress can induced RAC autophagy and promote RACs to regulate ECs function by releasing EVs.

#### 2.1.4 Retinal photoreceptors-derived EVs

Retinal photoreceptors (PRs) on the outer membrane of the retina are the most numerous and active cell in retina (28). It is certain that retinopathy is related to the thickness of the ratina and PRs (29). Maisto, R et al. (30) found that high glucose increased VEGF levels and decreased anti-angiogenic miR-20a-3p, miR-20a-5p, miR-106a-5p, and miR-20b expression either in PRs or in PR-derived EVs, RvD1 reverted the effects of glucose damage in photoreceptors and the pro-angiogenic potential of EVs, and ROS-induced NF-kB signaling was considered in relation to this mechanism. Therefore, inhibiting the changes of the outer membrane of the retina by regulating EVs may be a new treatment for the neovascularization of DR.

### 2.2 Extracocular cell-derived EVs

#### 2.2.1 Blood-derived EVs

##### 2.2.1.1 Serum/plasma-derived EVs

Biomarkers are very important indicators of abnormal biological conditions, and thus can be used as a powerful tool for early diagnosis of DR. Although biomarkers from ocular tissue are more reliable as pathological indicators, obtaining the sample of retina, vitreous fluid and aqueous humor is a high risk invasive procedure, whereas blood sample acquisition is relatively non-invasive, convenient and economical (31). Previous studies have confirmed that the biomarker function of blood-derived EVs could better identify DR at an early stage, and the changes in the quantity and content of EVs could also be used for assessing the severity of DR development.

By comparing the EVs and specific factors in serum or plasma of DR group, the diabetic group and the healthy control group, a large number of studies have found that the levels of cytokines and angiogenic factors in EVs of diabetic patients with retinopathy are significantly increased, which is correlated with the progression of diabetes (32). In addition, Mazzeo, A et al. (33) showed that the differences of miR-150-5p, miR-21-3p and miR-30b-5p in circulating EVs between diabetic patients and healthy subjects were significant, and decreased miR-150-5p and increased miR-21-3p, miR-30b-5p and HIF-1α may all together lead to vessel destabilization and angiogenesis. Zhang, Y et al. (34) found that the content of miR-26b-5p in serum-derived EVs of DR patients was up-regulated. Moreover, Xiao, J et al. (35) compared the proteomic profiles of plasma-derived small EVs from DR patients and normal subjects using iTRAQ-based quantitative proteomics. They found 90 proteins were significantly changed in DR, and confirmed that tumor necrosis factor-α-induced protein 8 (TNFAIP8) was upregulated in plasma-derived small EVs in the DR. All of these aforementioned molecules have been reported to be involved in endothelial dysfunction and angiogenesis, and may be consider as potential biomarkers for DR.

Other studies have found that plasma-derived EVs can be used as indicators of the stage of DR progression. Yu, B et al. (36) showed that miR-431-5p in plasma-derived EVs expression doubled in the PDR patients compared with the nonPDR patients and healthy controls, which indicate miR-431-5p could be used as a marker of disease entering the PDR stage. Tokarz, A et al. (37) suggested that the severity of DR was also statistically correlated with CCR5-positive plasma-derived large EVs.

Furthermore, several studies have shown that diabetic plasma-derived EVs were responsible for activation of the complement cascade. Huang, C et al. (38) found that the increased number of plasma-derived EVs in diabetic patients activated the complement system and upregulated of pro-inflammatory cytokines and chemokines, resulting in vascular injury. Through further research, they demonstrated that complement activation by IgG-laden EVs leads to membrane attack complex (MAC) deposition, promoting endothelium damage and DR progression (39).

##### 2.2.1.2 Platelet-rich plasma-derived EVs

EVs derived from platelet-rich plasma (PRP) transferred active agents that mediate intercellular crosstalk through phagocytosis, macropinocytosis, internalization, and endocytosis, affecting hemostasis and inflammatory responses (40). In addition, Zhang, W et al. (41) found that PRP-derived EVs mediated hyperglycemia-induced retinal endothelial injury through the TLR4/CXCL10 axis. Zhang, W et al. (42) demonstrated that DM-PRP-derived EVs activated YAP and enhanced müller cell proliferation and fibrosis through the PI3K/Akt pathway, thereby aggravating the PDR process and threatening the vision of patients.

#### 2.2.2 Pancreatic β-cells-derived EVs

Recent studies have shown that miRNAs released by pancreatic β-cells-derived EVs under diabetic conditions have effects on self and recipient cells through cell-to-cell communication (43). Kamalden, T.A et al. (44) showed that miR-15a was produced in pancreatic β-cells and transported in EVs to distant microvascular beds. As diabetes progresses, more EVs containing miR-15a entered into the blood stream, which leads to the development of diabetic microvascular complications including DR. They further showed that miR-15a-enriched EVs released from β-cells could be taken up by müller cells, causing oxidant stress and apoptosis *via* PI3-kinase signaling pathway.

#### 2.2.3 Lymphocyte-derived EVs

**Figure 1:**
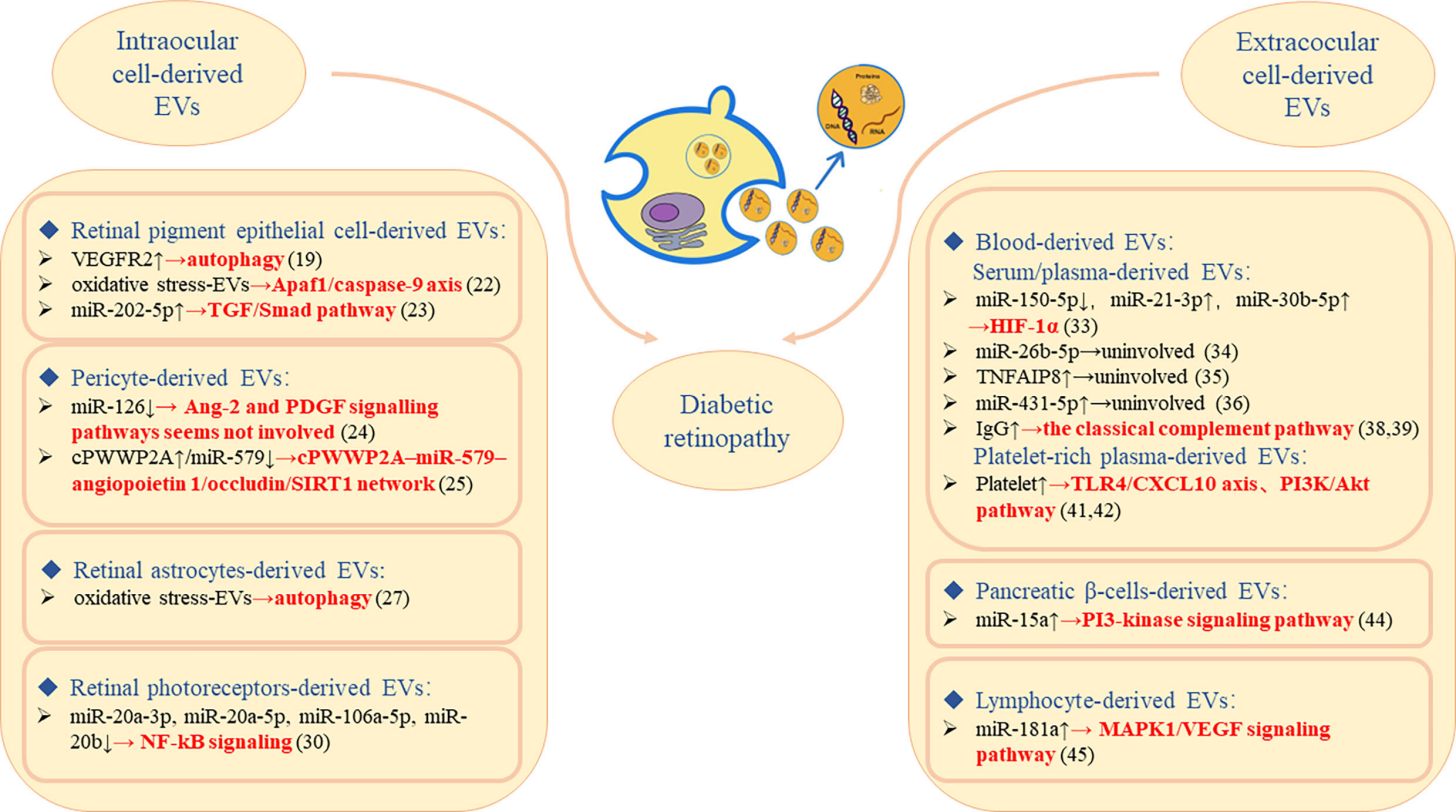
The molecular mechanism of DR regulated by intraocular/extraocular cell-derived EVs plays roles in the pathogenesis/diagnosis/prognosis of DR.

Yang, C et al. (45) found that miR-181a was selectively enriched in lymphocyte-derived EVs, and its overexpression could reduce EC viability and inhibit angiogenesis. They further confirmed *in vitro* and *in vivo* that miR-181a may play a role in reducing retinal neovascularization by interfering with the MAPK1/VEGF signaling pathway, which might provide a new therapeutic strategy for neovascularization (**Figure 1**).

## 3 EVs that play roles in treatment of DR

### 3.1 Mesenchymal stem cells-derived EVs

Mesenchymal stem cells (MSCs), which have the regeneration and differentiation ability and stability, can provide new therapeutic options for retinal diseases (46, 47). Moreover, it is gradually being confirmed that the paracrine trophic effect of MSCs can also be used in the treatment (48). EVs released from MSCs can not only provide the same effect as MSCs, but also avoid the side effects of cell transplantation therapy. Recent researches have demonstrated that MSC-derived EVs from different sources could repair DR-induced pathological changes of endothelial, müller and RPE cells, and played beneficial roles in the treatment of DR.

#### 3.1.1 Adipose tissue MSC-derived EVs

Gu, C et al. (49) applied EVs collected from adipose tissue derived MSC (AD-MSC) to human retinal microvascular endothelial cells, müller cells and RPE cells. They found that AD-MSC-derived EVs could relieve inflammation and angiogenesis by shuttling miR-192, which targeted and negatively regulated ITGA1, thereby reducing diabetic retinal damage. Safwat, A et al. (50) found the decreased expression level of micRNA-222 in retinal tissue of diabetes caused by STZ were associated with severe retinal injury and hemorrhage in different layers of retina and they observed the retinal repair effect of AD-MSC-derived EVs in streptozotocin-induced DM rabbit model by different routes including intravenous injection, subconjunctival injection and intraocular injection. They showed that AD-MSC-derived EVs were able to repair DM-induced retinal damage and hemorrhage by shuttling micRNA-222, which may occur through its role in regulation of vascular cell biology. And it is necessary to further explore the mechanism by which hyperglycemia caused decrease in expression level of micRNA-222.

#### 3.1.2 Umbilical cord MSC-derived EVs

Xu, N.D et al. (51) showed that human umbilical cord mesenchymal stem cells (UC-MSC)-derived EVs could modulate the proliferation, apoptosis and migration of human RPE cells in hypoxia. Furthermore, Zhang, W et al. (52) found that UC-MSC-derived EVs overexpressing miR-126 could attenuate hyperglycemia-induced inflammatory response and endothelial injury by inhibiting the HMGB1 signaling pathway. Moreover, Li, W et al. (53) injected UC-MSCs-derived EVs into the vitreous body of diabetic mouse model, and reveal that UC-MSCs-derived EVs decreased the levels of blood glucose and HbAlc, reduced the contents of inflammatory cytokines and VEGF, alleviated oxidative damage and inhibited retinal cell apoptosis in DR mice *via* shuttling miR-17-3p targeting STAT1.

#### 3.1.3 Bone marrow MSC-derived EVs

**Figure 2:**
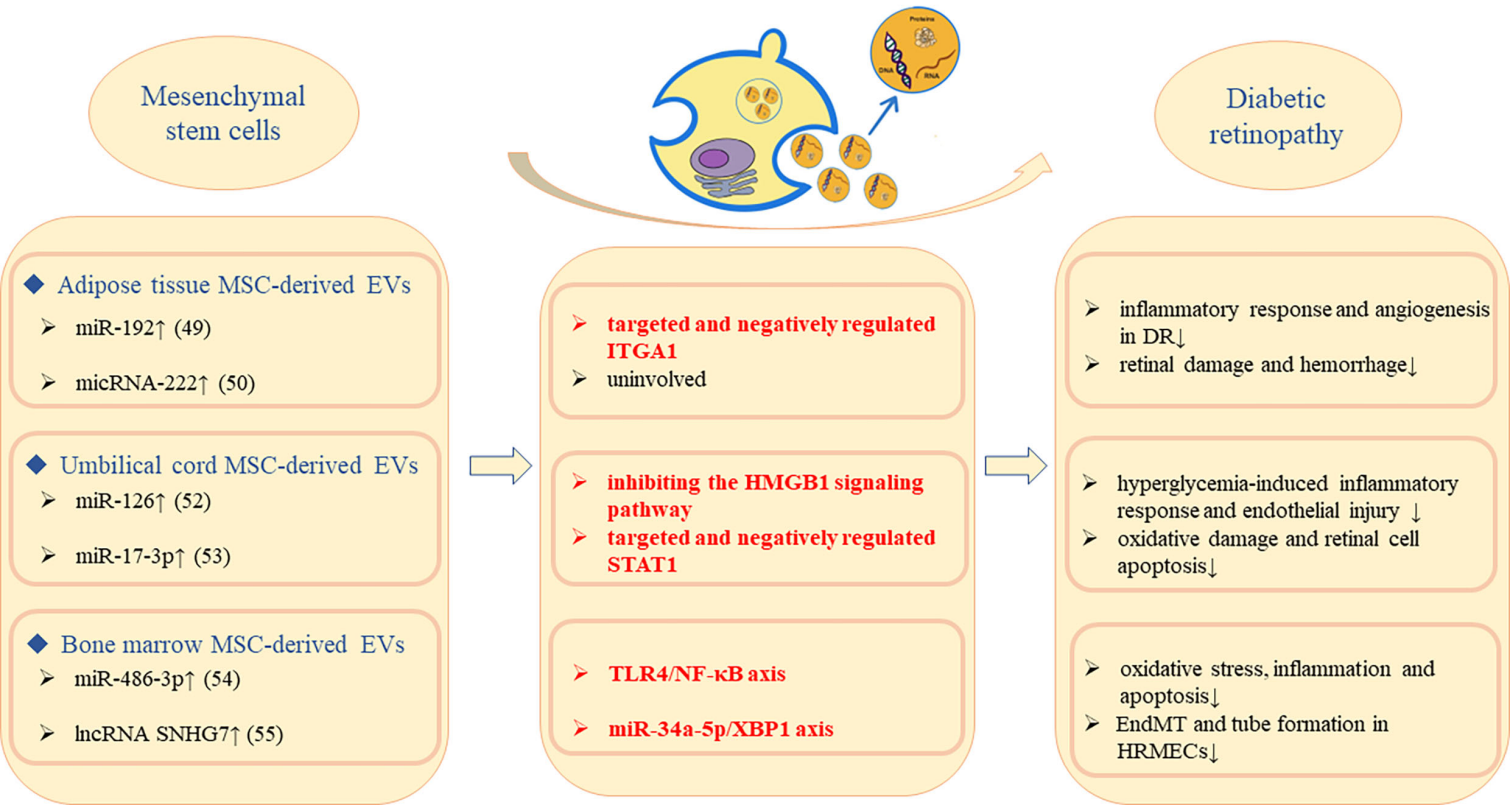
Molecular mechanism of MSC-derived EVs to alleviate DR progression.

Bone marrow contains the highest concentrations of adult stem cells, which are easily harvested and expanded in tissue culture and have the ability to repair damaged tissue, making them more promising for treating retinal diseases. Li, W et al. (54) made high glucose-treated müller cells co-cultured with bone marrow mesenchymal stem cells (BM-MSC)-derived EVs, and demonstrated that up-regulation of miR-486-3p induced by BM-MSC-derived EVs inhibited oxidative stress, inflammation and apoptosis *via* TLR4/NF-κB axis repression. Cao, X et al. (55) confirmed that BM-MSC-derived EVs could transfer SNHG7 to HRMECs and suppressed EndoMT and tube formation *via* miR-34a-5p/XBP1 axis. Both studies indicated that BM-MSC-derived EVs played protective roles in DR (**Figure 2**).

## 4 Limitations of EVs in DR

EVs can be used not only as diagnostic biomarkers for DR, but also as novel targets for therapeutic intervention in DR. Nonetheless, EVs has not been quantitatively used to grade the severity of DR, and EVs’ efficient and natural drug carrier role has not yet been used. The regulation of DR by EVs is very complex, and the protein and miRNA information carried by EVs still needs to be supplemented. The complete information database carried by EVs can provide directions for future researches of DR from pathogenesis to diagnosis and treatment.

## 5 Conclusion

The proteins and miRNAs contained in EVs can participate in the occurrence of DR pathological processes such as inflammation, oxidative stress, apoptosis, neovascularization and EndoMT through different mechanisms. Intraocular cell-derived EVs have two-sided effects according to the organism state. In physiological state, EVs maintain retinal homeostasis, while in pathological state, EVs induce oxidative stress and inflammation to damage retinal tissue, which can be alleviated by interfering with specific miRNAs or proteins in EVs. MSC-derived EVs can not only perfectly replicate the regeneration and differentiation abilities of MSCs, but also avoid their side effects. Therefore, EVs have the potential to be the natural material for the treatment of DR. Circulating EVs are relatively convenient and non-invasive to obtain, and the changes in their quantity and content of specific components in the early stage of DR are expected to be potential biomarkers for early diagnosis. In addition, circulating EVs can also regulate the neovascularization and inflammatory response of DR, which are expected to play the roles of DR drug therapy carriers. In conclusion, the research on EVs in DR is still in its infancy, and has broad application prospects, which need to be further explored.

## Author contributions

Conception or design: LF, FW. Search and organize literature: TC, JW. Drafting the work or revising: TC. Final approval of the manuscript: LF.
